# Good cop, bad cop? How (in-)consistencies in dual leadership shape employee strain and commitment

**DOI:** 10.3389/fpsyg.2026.1638116

**Published:** 2026-04-17

**Authors:** Laura Bartz, Jörg Felfe

**Affiliations:** 1 Criminological Research Institute of Lower Saxony, Hanover, Germany; 2 Work and Organizational Psychology, Helmut-Schmidt-University, Hamburg, Germany

**Keywords:** abusive supervision, commitment, dual leadership, leadership inconsistency, strain, transformational leadership

## Abstract

Although previous research has mostly examined positive and negative leadership separately, or less often within the same leader, little is known about how these leadership styles interact within dual leadership structures when subordinates have two leaders and how their (in-)consistency affects employees. This cross-sectional study with *N* = 2,602 employees from the German Federal Armed Forces examines profiles and interactions of abusive and transformational leadership from two leaders (i.e., an upper and a lower leader) and explores their relationship with employee strain and commitment. Five distinct dual-leadership patterns were identified: (1) a consistent positive profile, (2) a moderate positive profile, (3) a consistent negative profile, and (4) two inconsistent profiles. Employees in the consistently positive profile reported the lowest strain and highest commitment levels, while there were only rare differences in strain and commitment between the other profiles. Moreover, neither high levels of transformational leadership nor low levels of abusive leadership from a lower-level leader were sufficient to counteract the negative impact of an upper-level leader on employees. Instead, negative leadership from one leader undermines the effects of positive leadership of another leader. Although the cross-sectional design limits causal inferences, the reliance on self-reported data may introduce common method bias, and the specific organizational context may restrict generalizability, the findings highlight the importance of aligned leadership behavior when someone reports to two leaders simultaneously to foster employee strain and commitment. Importantly, dual leadership can only have positive effects on employees if both leaders pull in the same positive direction, as one leader cannot compensate for the negative behavior of the other. By integrating transformational leadership and abusive supervision within a dual leadership framework, this study advances leadership research by examining patterns of consistent and inconsistent leadership across two leaders.

## Introduction

In many organizational contexts, employees do not report to a single, uniform source of leadership but are exposed to multiple leaders who may convey convergent or divergent behavioral signals. To date, most studies have examined positive (i.e., addressing employee values and needs; Bass, 1998) and negative leadership (i.e., hostile verbal and non-verbal behavior towards employees; Tepper, 2000) in isolation and under the implicit assumption that leadership is relatively stable and consistent (e.g., Tautz et al., 2024; Zanchetta et al., 2026). However, more recent research focuses on leadership inconsistencies (Chénard-Poirier et al., 2022; Schilling et al., 2023; van Gerven et al., 2026) pointing out that not only negative behavior poses a threat to employees, but that also inconsistencies can put employee health at risk (Klebe et al., 2022; Klug et al., 2019).

Employees’ day-to-day experiences may be shaped not only by the presence of positive or negative leadership, but also by the (in-)consistency of leadership behaviors across different leaders. Dual leadership is a case in which employee health and commitment could be particularly at risk due to inconsistencies between different leaders. In practice, employees often report to two leaders with yet distinct but complementary roles and responsibilities that may either align or diverge (Ebbers and Wijnberg, 2017). For example, in a corporate setting, a department head (i.e., upper leader) oversees strategic alignment, budget approvals, and stakeholder communication, while a project manager (i.e., lower leader) is responsible for day-to-day execution, team coordination, and deadlines. Similarly, in a military context, a company commander (i.e., upper leader) may focus on tactical decisions, trainings objectives, and overall unit command, whereas a company sergeant major (i.e., lower leader) is responsible for discipline, personnel management, and daily operations.

Despite the prevalence of dual leadership in practice, research on dual leadership remains scarce. While some studies suggest correlations between the behavior of dual leaders (Bai et al., 2012; Litano and Morganson, 2020; Vidyarthi et al., 2014), dual leaders do not always act in harmony (Sahlmueller et al., 2022). Therefore, it is conceivable that different patterns of dual leadership exist, including *consistent positive* (i.e., high transformational and low abusive leadership for both leaders), *consistent negative* (i.e., low transformational and high abusive leadership for both leaders), or *inconsistent behavior* (i.e., varying degrees of transformational and abusive leadership for both leaders). Each of these patterns likely influences employee strain and commitment in distinct ways, underscoring the need for further research into the complex dynamics of dual leadership.

Particularly, it is important to know whether one positive leader can effectively mitigate the negative effects of another leader, or whether the effects of one positive leader are undermined by the negative behavior of another leader. It is particularly questionable whether a non-abusive or transformational lower-level leader can offset the damaging impact of a highly abusive upper-level leader, or whether the potential positive effect of a non-abusive or high transformational leader is diminished by a second leader who acts abusive. Prior research even suggests that leadership inconsistencies within the same leader may have more detrimental effects on employees than consistently negative behavior (Klebe et al., 2022; Ogunfowora, 2013; Schilling et al., 2023). For the case of two different leaders, a lower-level leader who merely refrains from abusive behaviors may offer only little relief, as inconsistencies can foster insecurity, increasing strain and reducing commitment. The same may apply for constellations where a highly abusive upper-level leader is paired with a transformational lower-level leader, as contrasting leadership dyads may send mixed signals so that the negative behavior of an abusive leader may overshadow any positive effect of another leader. Research has shown that variability in leaders’ fairness can be even more stressful for employees than consistently unfair treatment (Matta et al., 2017), so that employees may question their abilities and experience strain and disengagement (Schilling et al., 2023).

However, research on the effects of differing leadership styles and levels is yet outstanding. To address this research gap, the present study aims to explore heterogeneity and inconsistency in dual leadership. The first objective is to identify distinct dual leadership profiles based on the abusive and transformational leadership behaviors of two leaders and to examine their implications for employee strain and commitment. The second objective is to investigate how interactions of abusive and transformational behavior in dual leadership influence these outcomes. This study is the first to explore the extent to which inconsistencies between dual leaders affect employees and whether a second leader can buffer the negative consequences of poor leadership from another, or whether one bad leader undermines the positive effect of a good leader. First, it advances the yet scarce body of research on the impact of dual leadership on employees (Bai et al., 2012; Litano and Morganson, 2020; Sahlmueller et al., 2022; Vidyarthi et al., 2014) by examining leadership profiles and interactions between abusive and transformational leadership. Second, the study extends research on inconsistent leadership, primarily focused on intra-individual inconsistencies within the same leader (e.g., Chénard-Poirier et al., 2022; Klebe et al., 2022; Klug et al., 2019), by providing insights into inter-individual leadership inconsistencies between two leaders.

### Transformational, abusive, and inconsistent leadership

Leadership plays a crucial role in shaping employee well-being, influencing both positive and negative workplace experiences (Kaluza et al., 2021). Among the most widely studied leadership styles is transformational leadership, which inspires employees by addressing their values and higher-order needs, motivating them to exceed expectations (Montano et al., 2017). Transformational leadership comprises four key components: (1) *idealized influence*, where leaders serve as professional and ethical role models, (2) *inspirational motivation*, in which they provide a compelling vision that energizes employees, (3) *individualized consideration*, demonstrating support and recognition for employees’ needs, and (4) *intellectual stimulation*, which encourages innovation and challenges conventional approaches (Bass, 1998). Research has consistently shown positive effects of transformational leadership on employees, enhancing both their health and commitment (e.g., Eliyana et al., 2019; Franke and Felfe, 2011; Jiatong et al., 2022; Munir et al., 2012).

In contrast, destructive leadership encompasses both passive and actively harmful behaviors (Kaluza et al., 2019). A particularly damaging form is abusive supervision, where employees perceive their leader as engaging in hostile verbal and non-verbal behavior, such as public criticism, rudeness, or anger, without physical aggression (Tepper, 2000). Being linked to increased levels of emotional exhaustion, burnout, strain, and work–family conflict, this leadership style has, in contrast to transformational leadership, profound negative consequences for employee health and commitment (e.g., Alsadaan and Alqahtani, 2024; Hong et al., 2024; Yu et al., 2016).

The current study is primarily grounded in leadership theory, which often assumes leadership to be stable and consistent (e.g., Tautz et al., 2024; Zanchetta et al., 2026). However, some scholars suggest that leadership behaviors can vary across individuals, situations, and over time (de Cremer, 2003; Schilling et al., 2023). For example, some studies have focused on within-person fluctuations in leadership, demonstrating that leaders can exhibit varying or even contradictory behaviors (Duffy et al., 2002; Schilling et al., 2023). For instance, research has identified discrepancies between self- and employee-oriented leadership (Klug et al., 2019), variations under calm versus stressful conditions (Klebe et al., 2022), and even daily fluctuations in behaviors (Breevaart et al., 2014; Breevaart and Zacher, 2019). Although emerging research on inconsistent leadership has begun to question this stability assumption (e.g., Schilling et al., 2023; van Gerven et al., 2026), it has largely focused on fluctuations within the same leader. Extending this perspective, we shift the focus to inter-individual (in-)consistencies within dual leadership structures and examine how two leaders jointly shape employee experiences. While the present study is primarily grounded in leadership theory, the examined leadership behaviors can also be interpreted in light of broader stress-related frameworks. In this regard, abusive leadership may function as a social job demand, whereas transformational leadership may represent a social resource. At the same time, rather than fully adopting a general demand-resource framework, the present study focuses more specifically on the relational dynamics between two leaders and extends leadership research by examining inter-individual inconsistencies across leaders.

Dual leadership is given when employees report to two leaders with yet distinct but complementary roles and responsibilities that may either align or diverge (Ebbers and Wijnberg, 2017). The behavior of dual leaders is shaped by contextual and individual factors. Stressful working conditions, high workload, time pressure, or organizational change may limit leaders’ capacity to coordinate and enact consistent behaviors. Structural features such as unclear role boundaries or competing strategic and operational demands may further foster divergent leadership approaches. In addition, individual differences between leaders, differences in workload, or task complexity can influence how leadership behaviors are enacted and perceived, contributing to perceived inconsistency between two leaders.

In dual leadership structures, such divergence may confront employees with persistent yet contradictory relational signals. Drawing on paradox theory (Miron-Spektor et al., 2018; Smith and Lewis, 2011), inconsistent leadership can be understood as a paradoxical tension in which employees are simultaneously exposed to competing expectations that cannot easily be reconciled. For example, one leader may emphasize empowerment and support, while another relies on rigid control or even hostility. Because both leaders occupy legitimate authority positions, employees cannot simply ignore one set of signals but must cognitively and emotionally navigate these competing demands. These tensions may manifest as role ambiguity and value conflict, two well-established stressors in occupational stress research. Conflicting expectations regarding priorities, behavioral standards, or performance criteria create role ambiguity (i.e., unclear expectations about appropriate behavior) and value-related tension, thereby increasing psychological uncertainty. Managing such discrepancies requires continuous sensemaking and self-regulation, thereby increasing cognitive load and depleting psychological resources. Over time, this ambiguity may elevate emotional strain and weaken organizational commitment.

Conversely, positive consistent leadership across hierarchical levels may function as a valuable resource. When leaders convey coherent priorities and aligned behavioral standards, employees experience clarity and predictability, which facilitate orientation and reduce regulatory effort. Such consistency supports psychological stability and fosters well-being and positive organizational attitudes (Klebe et al., 2022; Klug et al., 2019). However, consistency is not inherently beneficial. If leadership across hierarchical levels is consistently negative or abusive, consistency may reinforce rather than mitigate harmful dynamics, potentially normalizing destructive behavior and exacerbating strain and disengagement over time.

However, the impact of inconsistencies within dual leadership, where one leader demonstrates supportive behavior while the other engages in destructive leadership, remains largely unexplored. Understanding these dynamics is crucial, as inconsistencies in leadership may introduce ambiguity, unpredictability, and potential strain for employees (Schilling et al., 2023; Vidyarthi et al., 2014), and influence each other in terms of compensation or buffering effects, ultimately shaping their well-being and commitment.

### Profiles of dual leadership and their associations with employee strain and commitment

Leadership research has traditionally conceptualized transformational and abusive leadership as individual-level constructs, focusing on behavioral consistency or ambivalence within a single leader (Klebe et al., 2022; Klug et al., 2019). However, employees in organizations are often simultaneously exposed to more than one formal leader. Extending established leadership theories to this interindividual level suggests that constructive and destructive leadership behaviors may not only coexist within persons but also be distributed across persons occupying different hierarchical roles and acting independently. From a configurational perspective, leadership can therefore be understood as a constellation of behaviors emerging across multiple leaders jointly influencing the same employees.

Research on dual leadership suggests that leaders often—but not always—move in the same direction (Bai et al., 2012; Litano and Morganson, 2020; Mayer et al., 2009; Sahlmueller et al., 2022; Vidyarthi et al., 2014). It seems plausible that leadership dynamics can vary, with some leader duos demonstrating alignment while others diverge in their approaches. While some studies in accordance with Social Learning Theory (Bandura, 1977) showed trickling down effects of transformational or abusive leadership (e.g., Bormann and Diebig, 2021; Mawritz et al., 2012), a recent study by Sahlmueller et al. (2022) highlighted discrepancies in employee perceptions of individual leadership between dual leaders. Specifically, employees rated the leadership effectiveness of regional leaders (i.e., upper-level leaders) as lower compared to that of business unit leaders (i.e., lower-level leaders). The same principle may apply to transformational and abusive leadership, where different constellations of dual leadership may emerge, ranging from consistent to inconsistent constellations.

For example, an upper-level leader who serves as a positive role model could influence a lower-level leader to adopt similar supportive and empowering behaviors (Bormann and Diebig, 2021; Mayer et al., 2009; Wang et al., 2018). In this scenario, both leaders would foster a work environment where employees feel valued, safe, and recognized, with minimal or no exposure to negative behaviors. This consistency in positive leadership is expected to be particularly beneficial for employee well-being (de Cremer, 2003; Schilling et al., 2023). Conversely, studies have also shown that negative leadership behaviors can trickle down from upper to lower leadership (Mawritz et al., 2012; Rice et al., 2021; Taylor et al., 2019). If an upper-level leader exhibits abusive supervision, a lower-level leader may adopt similar destructive behaviors, leading to a toxic work environment where employees feel unsafe and unappreciated. This constellation would create an atmosphere of stress and tense, undermining employee health and commitment.

However, not all leadership constellations follow a strictly positive or negative trajectory (Sahlmueller et al., 2022; Vidyarthi et al., 2014). Some leader duos may exhibit inconsistencies, where one leader displays transformational behavior such as motivating employees, addressing their needs, and refraining from abusive behavior, while the other leader displays hostile or unsupportive behavior, such as quick temper and lack of employee recognition. This misalignment may generate uncertainty and distress (Schilling et al., 2023; Vidyarthi et al., 2014), leaving employees without support and stability.

To date, transformational and abusive leadership have often been examined in isolation (Klebe et al., 2022), while research on dual leadership dynamics remains limited. Little is known about the prevalence and structure of diverse dual-leadership patterns, despite their relevance to employees’ day-to-day experiences. The aim is to capture both consistency and inconsistency in dual leadership by identifying distinct leadership profiles based on combinations of transformational and abusive leadership behaviors at two hierarchical levels (i.e., lower and upper leader). Identifying such leadership profiles provides a deeper understanding of their impact on employee outcomes.

Thus, integrating convergence mechanisms derived from Social Learning Theory with divergence mechanisms rooted in role differentiation provides a theoretical basis for expecting both consistent and inconsistent dual-leadership constellations. Taken together, this framework suggests that both consistent and inconsistent dual-leadership profiles are theoretically plausible. Given that leadership styles are often correlated (Bormann and Diebig, 2021; Mawritz et al., 2012; Mayer et al., 2009; Rice et al., 2021), we expect to find at least two consistent leadership profiles: (1) *a positive profile*, where both upper-level and lower-level leaders exhibit high transformational and low abusive leadership and (2) *a negative profile*, where both leaders display high abusive and low transformational leadership. However, in line with prior assumptions (Sahlmueller et al., 2022; Vidyarthi et al., 2014), we also anticipate at least (3) *two inconsistent leadership profiles*, where the upper-level leader exhibits high abusive and low transformational leadership, while the lower-level leader demonstrates the opposite pattern and vice versa. Based on these expectations, we derive the following hypothesis:

*Hypothesis 1:* Distinct profiles of dual leadership can be identified based on transformational and abusive leadership, including consistent profiles, characterized by (a) high scores for both upper-level and lower-level leader regarding transformational and low scores for abusive leadership, (b) low scores for both upper-level and lower-level leader regarding transformational and high scores for abusive leadership, and (c) inconsistent profiles, characterized by discrepancies between upper-level and lower- level leader regarding transformational and abusive leadership.

### Associations with employee strain and commitment

Since different constellations of transformational and abusive behavior in dual leadership structures reflect varying combinations of demands and resources for employees (Bakker and Demerouti, 2007), distinct associations with employee strain and commitment are to be expected. Specifically, employees experiencing consistently positive leadership, characterized by low abusive and high transformational behavior from both leaders, are expected to exhibit lower strain and higher commitment levels compared to those in consistently negative leadership environments (i.e., high abusive and low transformational leadership).

Moreover, they are also likely to fare better than employees experiencing inconsistent leadership patterns. Drawing on the literature on leadership inconsistency (Klebe et al., 2022; Schilling et al., 2023; Vidyarthi et al., 2014), it is plausible that employees who work under one negative (i.e., high abusive and low transformational leadership) and another positive leader (i.e., low abusive and high transformational leadership) may report higher strain and lower commitment than those in a consistently positive leadership environment. In addition to the general detrimental effects of abusive leadership (Alsadaan and Alqahtani, 2024; Hong et al., 2024), these employees may experience ambiguity regarding their own performance and behavior, perceiving their leadership duo as less authentic and reliable (Vidyarthi et al., 2014). Inconsistency may diminish the benefits of transformational behavior, as conflicting messages from dual leaders may increase strain and undermine commitment.

Furthermore, building on previous research showing that inconsistent leadership behaviors can negatively impact employees (Klebe et al., 2022; Klug et al., 2019; Schilling et al., 2023), it seems likely that strain and commitment levels among employees in inconsistent leadership constellations do not significantly differ from those in consistently negative leadership constellations. Employees in inconsistent leadership settings are likely to experience uncertainty and psychological stress due to contradictory expectations and mixed signals from their leaders (Schilling et al., 2023). This ambiguity can lead to confusion about performance expectations, hinder decision-making, and reduce feelings of stability and support. In turn, employees in consistently negative leadership environments may at least develop clearer expectations regarding their work environment (Klebe et al., 2022). The absence of inconsistency, though harmful, could allow employees to adapt to a predictable, albeit negative, work atmosphere. Consequently, the negative effects of unpredictability in inconsistent constellations may be just as detrimental to employee health and commitment as the exposure to constant negative leadership. Based on these considerations, we propose the following:

*Hypothesis 2a:* Employees in the consistently positive profile report lower strain and higher commitment than those with a consistently negative profile.

*Hypothesis 2b:* Employees in the consistently positive profile report lower strain and higher commitment than those with an inconsistent profile.

*Hypothesis 2c:* Employees in the inconsistent profile report similar levels of strain and commitment as those with a consistently negative profile.

### Interactions within dual leadership

Abusive leaders displaying hostile behavior and a lack of recognition endanger employee well-being and commitment. To mitigate these negative effects, prior research has examined various moderating factors that can buffer the detrimental consequences of abusive supervision. Studies have identified protective factors, such as workplace friendship (Arshad et al., 2021), co-worker support (Hobman et al., 2009; Pradhan and Jena, 2018), organizational support (Kim et al., 2015; Li et al., 2016), political skills (Li et al., 2016), organizational justice (Lee et al., 2018), autonomy (Velez and Neves, 2016), or individual resilience (Jahedian et al., 2021). These resources can help employees in coping with harmful effects of abusive leadership by providing social and emotional support or fostering control and fairness in the workplace.

To date, little is known about the role of a second leader in a dual leadership setting. In dual leadership systems, two leaders could either operate independently or interact in shaping employee well-being. Independence would imply that the behaviors of the upper- and lower-level leaders have no reciprocal influence on each other. However, this seems unlikely as dual leaders typically fulfill complementary roles and share responsibilities (Ebbers and Wijnberg, 2017). It remains open whether a second leader, who either demonstrates high transformational or at least low abusive leadership, can effectively mitigate the harmful effects of another abusive leader, or whether the effect of one positive leader is neutralized if the other leader behaves negatively. Similar to the second proposition, in this inconsistent case negative effects of one leader may not be buffered by the other, but the positive effect of one leader may be neutralized by the other. Schilling et al. (2023) suggest that inconsistencies within leaders may have even more severe consequences for employees than consistent destructive leadership. Understanding this dynamic between dual leaders is essential for assessing how different leadership constellations affect employee strain and commitment.

### Interactions of abusive and transformational leadership

Transformational leaders actively engage in positive behaviors, leading to two possible interaction scenarios with abusive supervision. According to the JD-R model, it would be plausible that a transformational lower-level leader serves as a resource that helps to counterbalance the harmful effects of an abusive upper-level leader (Bakker and Demerouti, 2007, 2017; Schyns and Schilling, 2013; Wu and Hu, 2009), as transformational leaders provide support and motivation, creating a sense of trust and guidance that helps to reduce employee strain and to foster commitment (Breevaart and Bakker, 2018). However, in the case of dual leadership resource and demand may be more closely intertwined and cannot influence each other independently. Given that dual leaders are unlikely to operate independently from each other, we propose that more positive leadership at one level (i.e., not being abusive) will only benefit employees when also the other leader demonstrates similar behavior as inconsistencies may create a tense work environment, where unclear expectations, ambiguities, and a need for self-protection elicit strain and disengagement among employees (Klebe et al., 2022; Ogunfowora, 2013; Schilling et al., 2023). Similarly, the more positive impact of one transformational leader may be undermined if the other leader displays abusive supervision due to contradictory expectations and communication. In a lab study, Matta et al. (2017) could show that variably fair treatment of leaders resulted in even more stress than consistently unfair treatment. In a dual leadership context, differences in leadership styles (i.e., abusive vs. transformational leadership) could thus lead to confusion and insecurity, fostering employee strain and diminishing commitment. For example, a transformational leader may encourage employees to challenge the status quo and come up with new ideas (intellectual stimulation). If the other leader questions the competence of the employees and threatens negative consequences for mistakes or stupid suggestions, the employees will not dare to follow the request of the transformational leader.

While employee strain is expected to be the lowest and commitment the highest when the upper-level leader exhibits low abusive, and the lower-level leader high transformational leadership, strain is expected to be the highest and commitment the lowest when the upper-level leader is highly abusive, and the lower-level leader lacks transformational behaviors. However, in an inconsistent constellation where an upper-level leader shows abusive behavior while a lower-level leader displays transformational behavior (or vice versa), employees are likely to experience significant contradictions in leadership. Instead of fully benefiting from transformational leadership of one leader, employees may perceive a lack of cohesion, struggle with contrasting behaviors or expectations, and question their own abilities and competences (Schilling et al., 2023), diminishing the positive effects of transformational leadership of the other leader. For example, when a lower-level leader aims to motivate employees by supportive and inspirational behaviors, but the upper-level leader frequently belittles and humiliates employees and their performance, these conflicting and negative messages may undermine any positive effect of transformational behavior.

Accordingly, we anticipate only small differences in employee outcomes when comparing consistently negative leadership duos (i.e., high abusive supervision and low transformational behavior) with inconsistent duos (i.e., high transformational and high abusive behavior). In such cases, the presence of abusive leadership is expected to create a negative climate that overshadows the benefits of transformational behavior, resulting in similar strain and commitment levels in both constellations. Conversely, we expect more pronounced differences when comparing inconsistent leadership duos with consistently positive duos (i.e., low abusive and high transformational behavior), as a positive combination likely creates a stable and trusting environment, enhancing employee commitment and reducing strain.

Following previous findings on leadership inconsistencies (Klebe et al., 2022; Matta et al., 2017; Schilling et al., 2023), we expect that the negative behavior of one leader (here: upper-level leader, leader) (1) may neutralize the benefits of a second positive leader (here: lower-level leader, leader) (2) so that dual leadership only fosters employee commitment and alleviate employee strain when both leaders engage in positive behaviors (i.e., low abusive and high transformational leadership). Accordingly, we expect only minor differences in employee strain and commitment between consistently negative and inconsistent leadership duos, as any exposure to abusive leadership likely diminishes the positive influence of transformational behavior. In contrast, consistently positive leadership is expected to yield the most favorable employee outcomes, with more pronounced differences to inconsistent leadership constellations. We expect the following:

*Hypothesis 3a:* Transformational leadership of leader 1 interacts with abusive leadership of leader 2 regarding employee strain and commitment. Positive leadership in terms of transformational leadership of leader 1 will only have positive effects on employees if leader 2 refrains from abusive behavior, but effects are neutralized, if leader 2 behaves abusively.

### Interactions of high and low abusive leadership

Regarding the interplay of abusive leadership from upper- and lower-level leaders, employee strain is expected to be the lowest and commitment the highest when both leaders avoid abusive behaviors and employee strain is expected to be the highest and commitment the lowest when both leaders highly engage in abusive behaviors (Ogunfowora, 2013; Schyns and Schilling, 2013; Wu and Hu, 2009). Another critical scenario may arise when dual leadership is inconsistent. It seems plausible that the interplay of one high and one low abusive leader follows a similar pattern to abusive and transformational leadership in terms of leadership inconsistency. Accordingly, inconsistent leadership elicits strain because unpredictable behaviors create uncertainty and stress (Klebe et al., 2022; Schilling et al., 2023). For example, when an upper-level leader consistently criticizes and ridicules employees in front of others, this behavior harms employees even when the lower-level leader refrains from such behaviors, neutralizing any positive effect from the lower-level leader. Instead of benefiting from low abusive behavior of one leader, employees may question the lack of support in this critical situation, increasing strain and lowering commitment.

Accordingly, we expect only small differences in employee strain and commitment when comparing constellations in which both leaders show high levels of abusive behavior (i.e., consistent negative) to those where only the upper-level leader is abusive (i.e., inconsistent). Regarding our example, strain and commitment levels due to constant criticism will be similar when only one or both leaders make offensive comments. In both cases, the presence of abusive behavior from one leader can create a negative leadership climate that overshadows any potentially positive effects from another less abusive leader. As a result, employees are likely to feel similarly strained and disengaged in both scenarios. In contrast, we expect more pronounced differences when comparing inconsistent leader constellations to constellations in which both leaders show only low levels of abusive behavior (i.e., consistent positive). A consistently non- or low abusive leadership duo across leadership levels can provide more psychological safety, stability, and trust, decreasing strain and increasing commitment.

This notion aligns with prior research on inconsistencies *within* individual leaders. For example, Ogunfowora (2013) showed that variability in abusive behaviors within leaders is associated with diminished perceptions of leader and organizational ethicality, reduced job satisfaction and commitment, and increased counterproductive work behaviors. Also, Klebe et al. (2022) showed that inconsistencies in abusive supervision account for additional variance in employee strain beyond consistent patterns as inconsistent leadership is unpredictable and difficult to anticipate for employees.

Therefore, it is expected that dual leadership can only have positive effects on employees, when both leaders pull in the same positive direction and show no or only low abusive behaviors. If one leader is abusive (here: upper-level leader, leader 1), the potential positive effect of the other non-abusive leader (here: lower-level leader, leader 2) is neutralized. Employee strain and commitment levels are expected to differ only slightly between constellations where both leaders versus only the upper-level leader display abusive behavior, as any presence of abuse can deteriorate the leadership climate and reduce the benefits of positive leadership. In contrast, consistent low abusive behavior across both leaders is likely to enhance psychological safety, leading to higher differences in strain and commitment compared to inconsistent constellations. We therefore hypothesize the following:

*Hypothesis 3b:* Abusive leadership of leader 1 interacts with abusive leadership of leader 2 regarding employee strain and commitment. Positive leadership in terms of lower abusive supervision of leader 1 will only have positive effects on employees if leader 2 also refrains from abusive behavior, but effects are neutralized, if leader 2 behaves abusively.

## Methods

### Sample and procedure

Data were collected between December 2014 and January 2015 using a questionnaire administered both online and via paper-and-pencil. Participants were recruited through various units of the armed forces that agreed to support the study and facilitated the distribution of the survey. The survey included measures of transformational and abusive leadership for both leaders, as well as employee stress and commitment as outcomes. Further variables (e.g., health and organizational citizenship behavior) were assessed but are not considered in the present study. In total, the survey consisted of 58 items covering all study variables. The number of items for each scale is indicated in the ‘Measures’ section. The sample consists of *N* = 2,602 employees of the German Federal Armed Forces. Participants indicated working either in the army (52.3%), the air force (4.8%), or the joint support and enabling services (i.e., logistical, operational, and administrative support; 42.4%). The majority of the sample were men (91.2%), and the average age was *M* = 33.3 years (*SD* = 8.44). However, it must be noted that only 9.1% of the participants indicated their age. Data were collected via paper-pencil surveys. The procedure was conducted in line with the ethical principles of the Declaration of Helsinki, ensuring voluntary participation, informed consent, confidentiality, and anonymity. The study was registered and approved by the Psychological Services of the German Federal Armed Forces (registration number 2/05/12).

### Measures

In the survey, employees reported transformational and abusive leadership for (1) their upper-level leader, which is the company commander who is responsible for tactical decisions and the overall unit command, and (2) their lower-level leader, which is the sergeant major who is responsible for daily organization, discipline, training and supply (“mother of the company”). Moreover, employees rated their own strain and affective commitment to the organization. Items for each of the scales were rated on a five-point Likert scale ranging from 1 = *not at all true* to 5 = *completely true*.

#### Transformational leadership

Transformational leadership of both leaders was reported with the German version of the Multifactor Leadership Questionnaire (MLQ, Felfe, 2006; originally by Bass and Avolio, 1995). Each subscale (i.e., idealized influence attributed, inspirational motivation, intellectual stimulation, individual consideration) was measured with three items. An example item is “My direct supervisor spends time coaching me”. Cronbach’s Alpha was *α* = 0.93 for transformational leadership of the upper-level leader and *α* = 0.94 for the lower-level leader.

#### Abusive supervision

Abusive leadership of both leaders was rated with the German version of the Abusive Supervision Scale (Loock and Schilling, 2010; originally by Tepper, 2000). Offensive-humiliating behaviors were measured with three items and insincere-unfair behaviors with one item. An example item is “It may occur that my direct supervisor humiliates employees in front of others”. Cronbach’s Alpha was *α* = 0.81 for abusive leadership of the upper-level leader and *α* = 0.83 for the lower-level leader.

#### Employee strain

Employee strain was measured as an indicator for their mental health. Employees rated their own strain with the Irritation Scale by Mohr et al. (2005) with three items for cognitive irritation and three items for emotional irritation. An example item is “*I have difficulty relaxing after work”.* Cronbach’s Alpha was *α* = 0.89.

#### Employee commitment

Employees’ organizational commitment was measured using the subscale affective organizational commitment of the COMMIT by Felfe and Franke (2012) according to Allen and Meyer (1990). Commitment was measured with three items, for example ‘*I feel a strong sense of belonging to my organization*’. Cronbach’s Alpha was *α* = 0.78.

#### Control variables

Because employee health may be influenced by gender and type of work, we controlled for participants’ gender and unit.

### Statistical analyses

To identify profiles of transformational and abusive leadership in dual leader constellations (Hypothesis 1), a latent profile analysis (LPA) was conducted. Profiles were calculated based on four leadership behaviors, that is, (1) transformational and (2) abusive leadership of the upper-level leader, as well as (3) transformational and (4) abusive leadership of the lower-level leader using the maximum likelihood procedure in Mplus 7 (Muthén and Muthén, 1998–2017). The number of profiles was determined based on the following criteria (Nylund et al., 2007): (1) model fit as indicated by the adjusted Bayesian Information Criterion (aBIC) and likelihood ratio tests (LMRT and VLMRT); (2) classification quality based on entropy and average latent class posterior probabilities (AvePP); (3) profile prevalence (no less than 1% of the sample in one profile), and (4) clarity and theoretical interpretability of the profiles. Lower values for aBIC indicate better model fit.

To test differences between the profiles regarding employee strain and commitment (Hypotheses 2a-c), ANCOVAs were calculated for each variable controlling for gender and unit. To test interactions between leadership behavior of the upper- and lower-level leader (Hypothesis 3a-b), moderation analyses were calculated, also controlling for gender and unit.

## Results

As shown in Table 1, leadership styles of both leaders were positively correlated (transformational leadership: *r* = 0.53, *p* < 0.001; abusive leadership: *r* = 0.53, *p* < 0.001). Moreover, both behaviors were associated with employee strain and commitment. Transformational behaviors of upper-level (*r* = −0.11, *p* < 0.001) and lower-level leader (*r* = −0.06, *p* < 0.01) were negatively related to employee strain and positively linked to employee commitment (*r* = 0.31, *p* < 0.001 for upper-level and *r* = 0.29, *p* < 0.001 for lower-level leader). In contrast, abusive behaviors from both the upper-level (*r* = 0.15, *p* < 0.001) and the lower-level leader (*r* = 0.13, *p* < 0.001) were positively related to employee strain and negatively to employee commitment (*r* = −0.19, *p* < 0.001 for upper-level and *r* = −0.20, *p* < 0.001 for lower-level leader).

**Table 1:** Means, standard deviations, and correlations for gender, age, transformational leadership of upper and lower leader, abusive leadership of upper and lower leader, employee strain and commitment.

No.	Study variables	M (SD)	1	2	3	4	5	6	7	8
1	Gender	-	-							
2	Age	33.30 (8.44)	−0.19**	-						
3	TFL UL	3.41 (0.91)	0.03	−0.11	(0.93)					
4	TFL LL	3.40 (0.95)	0.02	−0.04	0.53***	(0.94)				
5	AS UL	1.91 (0.90)	−0.01	0.12	−0.49***	−0.24***	(0.81)			
6	AS LL	2.02 (0.98)	−0.04	−0.02	−0.28***	−0.55***	0.53***	(0.83)		
7	Strain	2.39 (1.03)	0.08***	0.29***	−0.11***	−0.06**	0.15***	0.13***	(0.89)	
8	Commitment	3.63 (0.98)	−0.02	−0.02	0.31***	0.29***	−0.19***	−0.20***	−0.09***	(0.78)

*N* = 582. *** *p* < 0.001, ** *p* < 0.01, * *p* < 0.05. TFL = transformational leadership, AS = Abusive Supervision, UL = upper-level leader, LL = lower-level leader. Gender coded as 1 = male, 2 = female. Alpha coefficients across the diagonal in parentheses.

### Profiles of dual leadership

Table 2 presents the results of the latent profile analysis, comparing models from two to seven profiles. Based on fit criteria, parsimony, and interpretability, the five-profile solution was selected as the final model. The likelihood ratio tests were also significant for the two-, three-, six-, and seven-profile models. However, we opted for the more parsimonious five-profile solution, as the other profile models did not provide substantially distinct or meaningfully interpretable patterns by comparison. The five-profile solution demonstrated good classification quality in terms of entropy, average posterior probabilities (Ave PP), and profile proportions.

**Table 2:** Latent profile analyses of dual leadership.

No. of profiles	No. of free parameters	logL	aBIC	VLMRT	LMRT	Entropy	AVE PP	Latent profile counts / proportions
2	13	−12641.79	25344.35	0.000	0.000	0.777	0.91–0.95	1763 (68.55%)/809 (31.45%)
3	18	−12315.26	24714.68	0.000	0.000	0.794	0.86–0.92	891 (34.64%)/1,402 (54.51%)/290 (10.85%)
4	23	−12053.20	24213.93	0.311	0.317	0.822	0.89–0.93	1,385 (53.85%)/242 (9.41%)/779 (30.29%)/166 (6.45%)
**5**	**28**	**−11829.03**	**23788.96**	**0.000**	**0.000**	**0.862**	**0.86–0.94**	**1,370 (53.27%)/113 (4.39%)/146 (5.68%)/178 (6.92%)/765 (29.74%)**
6	33	−11654.44	23463.15	0.001	0.001	0.790	0.75–0.91	551 (21.42%)/151 (5.87%)/1,014 (39.43%)/592 (23.02%)/106 (4.12%)/158 (6.14%)
7	38	−11492.18	23162.01	0.001	0.001	0.796	0.77–0.88	423 (16.45%)/561 (21.81%)/335 (13.03%)/106 (4.12%)/149 (5.79%)/118 (4.59%)/880 (34.22%)

Final solution in boldface. logL = Log likelihood. aBIC = sample size-adjusted Bayesian Information Criterion. VLMRT = Vuong-Lo–Mendell likelihood ratio test. LMRT = Lo–Mendell likelihood ratio test. AvePP = average latent class posterior probabilities.

**Figure 1:**
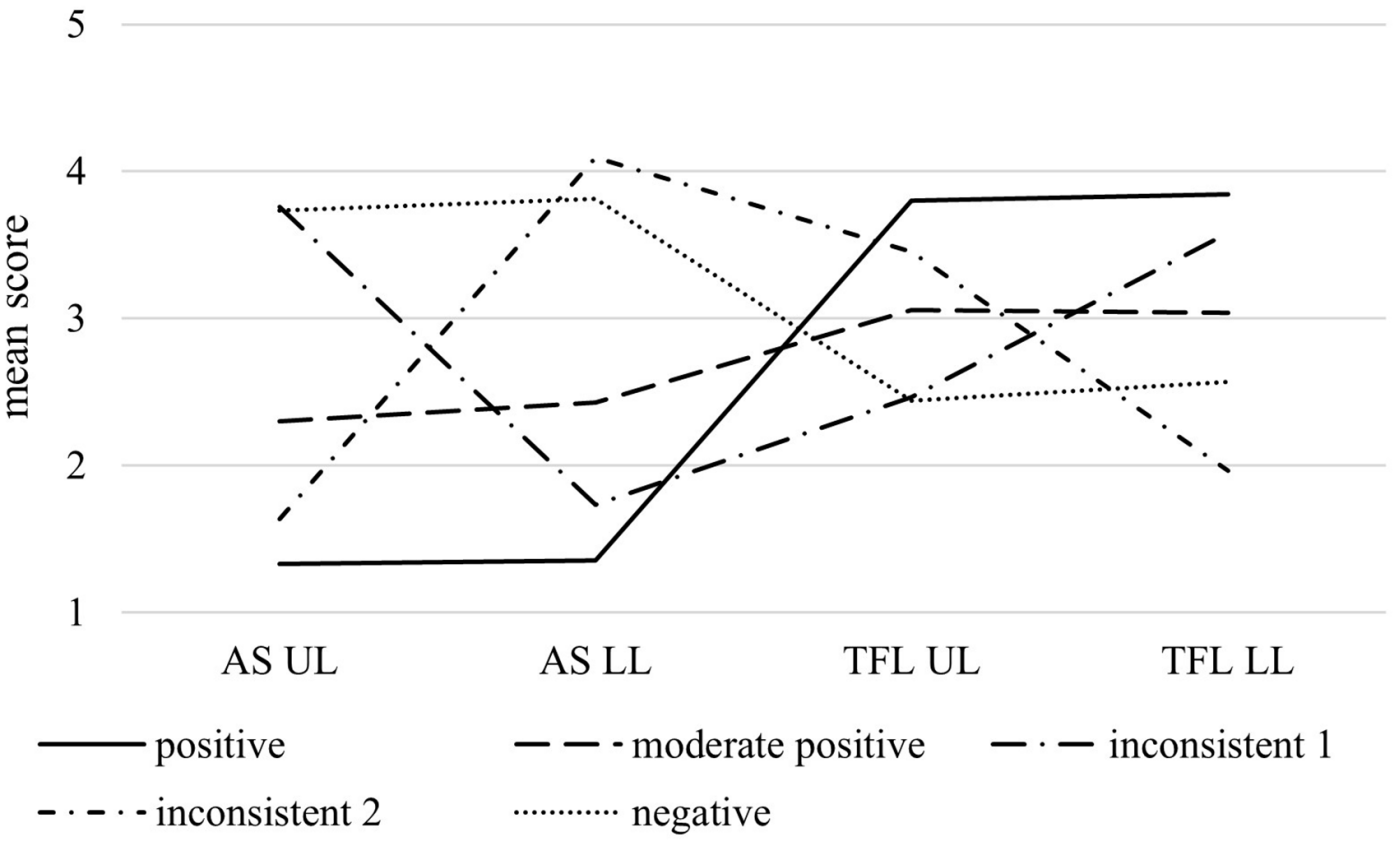
Profiles of dual leadership: mean scores of abusive supervision of upper-level (AS UL) and lower-level leader (AS LL), as well as transformational leadership of upper-level (TFL UL) and lower-level leader (TFL LL) across the five profiles of dual leadership.

Figure 1 depicts the five dual leadership profiles based on mean scores for all dual leadership dimensions. Table 3 summarizes the five dual-leadership profiles regarding their core characteristics as well as their differential relationships with strain and commitment. The first profile, labeled *positive* (53.27% of the sample), was characterized by low abusive and high transformational leadership from both upper-level and lower-level leaders. The second profile, labeled *moderate positive* (29.74% of the sample), displayed moderate to low abusive supervision and moderate transformational leadership for both leaders. The third profile, labeled *inconsistent 1* (4.39% of the sample), showed high abusive supervision and moderate to low transformational leadership from the upper-level leader, while the lower-level leader exhibited low abusive and high transformational leadership. The fourth profile, labeled *inconsistent 2* (5.68% of the sample), presented the opposite pattern, with low abusive supervision and moderate to high transformational leadership from the upper-level leader, but high abusive supervision and low transformational leadership from the lower-level leader. Finally, the fifth profile, labeled *negative* (6.92% of the sample), was marked by high abusive supervision and moderate to low transformational leadership from both leaders.

**Table 3:** Overview of dual leadership profiles, their characteristics, and associations with outcomes.

Profile	% of sample	Upper-level leader	Lower-level leader	Consistency pattern	Strain	Commitment
Positive	53.27%	Low abusive, high transformational	Low abusive, high transformational	Consistent positive	Lowest	Highest
Moderate positive	29.74%	Moderate-low abusive, moderate transformational	Moderate-low abusive, moderate transformational	Consistent Moderately positive	Moderate	Moderate
Inconsistent 1	4.39%	High abusive, moderate-low transformational	Low abusive, high transformational	Inconsistent (upper negative / lower positive)	Elevated	Intermediate
Inconsistent 2	5.68%	Low abusive, moderate-high transformational	High abusive, low transformational	Inconsistent (upper positive / lower negative)	Elevated	Low
Negative	6.92%	High abusive, moderate-low transformational	High abusive, moderate-low transformational	Consistent negative	Highest	Lowest

In summary, we identified (1) two consistent profiles – a consistent positive (i.e., low abusive supervision and high transformational leadership from both leaders) and a consistent negative profile (i.e., high abusive supervision and moderate to low transformational leadership from both leaders) – as well as (2) two inconsistent profiles (i.e., discrepancies in abusive and transformational leadership between upper- and lower-level leader). Additionally, we detected (3) one moderate positive profile (i.e., moderate to low abusive and moderate transformational leadership from both leaders). These findings support Hypotheses 1 a-c.

### Differences in strain and commitment across profiles

**Figure 2:**
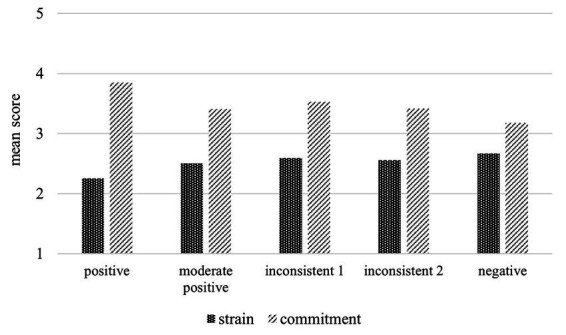
Mean scores of employee strain and commitment across the five profiles of dual leadership.

The ANCOVA results presented in Figure 2 provide an overview of the mean strain and commitment levels across all profiles. First, we hypothesized that employees in the *positive* profile would report lower strain and higher commitment compared to those in the *negative* profile. Results (i.e., adjusted for pairwise comparisons between the five profiles) indicate that employees in the *positive* profile reported lower strain (*M* = 2.25, *SD* = 0.99 vs. *M* = 2.67, *SD* = 1.12; *SE* = 0.08, *p* < 0.001) and higher commitment (*M* = 3.85, *SD* = 0.92 vs. *M* = 3.18, *SD* = 1.10; *SE* = 0.08, *p* < 0.001) than employees in the *negative* profile. Hypothesis 2a was supported.

Second, we expected employees in the *positive* profile to experience lower strain and higher commitment compared to those in the *inconsistent* profiles. Results confirm that employees in the *positive* profile reported lower strain (*M* = 2.25, *SD* = 0.99 vs. *M* = 2.59, *SD* = 1.08; *SE* = 0.10, *p* < 0.001) and higher commitment (*M* = 3.85, *SD* = 0.92 vs. *M* = 3.53, *SD* = 1.03; *SE* = 0.09, *p* < 0.05) than employees in the *inconsistent profile 1*. Similarly, employees in the *positive* profile reported lower strain (*M* = 2.25, *SD* = 0.99 vs. *M* = 2.56, *SD* = 1.16; *SE* = 0.09, *p* < 0.05) and higher commitment (*M* = 3.85, *SD* = 0.92 vs. *M* = 3.42, *SD* = 1.17; *SE* = 0.09, *p* < 0.001) than employees in the *inconsistent profile 2*. These findings support Hypothesis 2b.

Third, we hypothesized that strain and commitment levels in the *inconsistent* profiles would be similar to those in the *negative* profile. Results indicate that employees in the *inconsistent profile 1* reported similar strain levels (*M* = 2.59, *SD* = 1.08 vs. *M* = 2.67, *SD* = 1.12; *SE* = 0.13, *p* = 1.00), but higher commitment levels as in the *negative* profile (*M* = 3.53, *SD* = 1.03 vs. *M* = 3.18, *SD* = 1.10; *SE* = 0.12, *p* < 0.05). Moreover, employees in the *inconsistent profile 2* reported similar strain (*M* = 2.56, *SD* = 1.16 vs. *M* = 2.67, *SD* = 1.12; *SE* = 0.12, *p* = 1.00) and commitment levels (*M* = 3.42, *SD* = 1.17 vs. *M* = 3.18, *SD* = 1.10; *SE* = 0.11, *p* = 0.28) as employees in the *negative* profile. Thus, Hypothesis 2c was supported regarding differences in strain, but regarding commitment only when comparing the inconsistent profile 2 with the negative profile.

Additionally, employees in the *moderate positive* profile, reported higher strain (*M* = 2.50, *SD* = 1.03 vs. *M* = 2.25, *SD* = 0.99; *SE* = 0.05, *p* < 0.001) and lower commitment levels (*M* = 3.41, *SD* = 0.92 vs. *M* = 3.85, *SD* = 0.92; *SE* = 0.04, *p* < 0.001) than those in the *positive* profile, but did not differ from any other profile.

In summary, our hypotheses regarding differences in strain and commitment across profiles were largely supported. Significant differences emerged, with the *positive* profile displaying the most favorable outcomes and the *negative* profile the least favorable, while the inconsistent profiles showed similar levels of strain and partially similar levels of commitment compared to the negative profile.

### Interactions of dual leadership

First, we expected that the abusive leadership of one leader would interact with the transformational leadership of another leader. Results show a positive relationship between abusive leadership of an upper-level leader and employee strain (*B* = 0.16, *SE* = 0.02, *p* < 0.001), and a negative relationship between abusive leadership and employee commitment (*B* = −0.14, *SE* = 0.02, *p* < 0.001). However, the interaction between abusive leadership of an upper-level leader and transformational leadership of a lower-level leader were neither significant for employee strain (*B* = 0.02, *SE* = 0.02, *p* = 0.46, *ΔR^2^* = 0.000), nor for employee commitment (*B* = 0.00, *SE* = 0.02, *p* = 1.00, *ΔR^2^* = 0.000). Hypothesis 3a is thus rejected.

**Figure 3:**
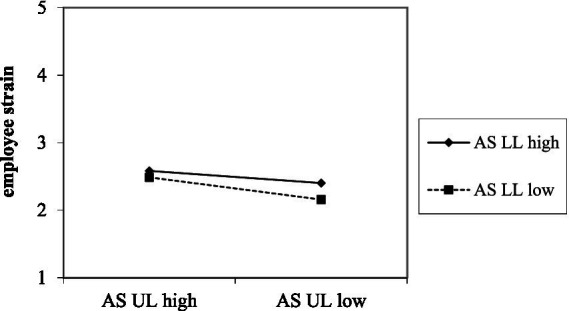
Interaction between upper-level (UL) and lower-level (LL) leaders’ abusive leadership (AS) on employee strain.

**Figure 4:**
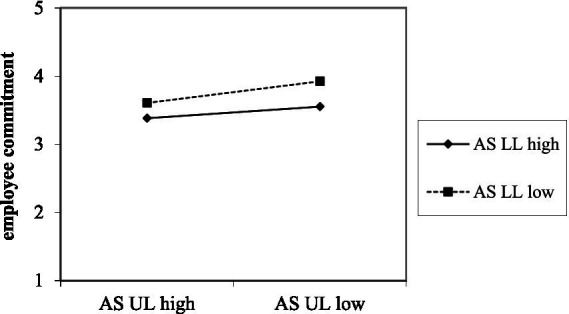
Interaction between upper-level (UL) and lower-level (LL) leaders’ abusive supervision (AS) on employee commitment.

Second, we expected that abusive leadership of one leader would interact with abusive leadership of another leader. Results show a positive relationship between abusive supervision of an upper-level leader and employee strain (*B* = 0.14, *SE* = 0.03, *p* < 0.001). As can be seen in Figure 3, low abusive supervision only affects employee strain negatively, when both leaders behave in the same manner, but this effect is neutralized, when one leader behaves highly abusive (*B* = −0.04, *SE* = 0.02, *p* < 0.05, *ΔR^2^* = 0.002). Simple slope analyses were significant for both high (*B* = 0.10, *SE* = 0.03, 95% CI [0.04, 0.16], *p* < 0.01) and low abusive supervision of the lower-level leader (*B* = 0.18, *SE* = 0.04, 95% CI [0.11, 0.26], *p* < 0.001). Moreover, results show a negative relationship between abusive supervision of an upper-level leader and employee commitment (*B* = −0.13, *SE* = 0.03, *p* < 0.001). As shown in Figure 4, low abusive behavior only affects employee commitment positively, when both pull in the same positive direction, but this effect is neutralized, when one leader behaves highly abusive (*B* = 0.04, *SE* = 0.02, *p* < 0.05). Simple slope analyses were significant for both high (*B* = −0.09, *SE* = 0.03, 95% CI [−0.15, −0.04], *p* < 0.01) and low abusive supervision of the lower leader (*B* = −0.18, *SE* = 0.04, 95% CI [−0.25, −0.10], *p* < 0.001). Hypothesis 3b is supported.

## Discussion

The objective of this study was to account for heterogeneity in employees’ experiences of dual leadership, specifically regarding its implications for employee strain and organizational commitment. By examining consistent and inconsistent leadership constellations, the study extends the understanding of dual leadership dynamics beyond the predominant focus on within-person variability (e.g., Breevaart et al., 2014; Klebe et al., 2022; Klug et al., 2019; van Gerven et al., 2026). It is the first study to identify profiles of dual leadership that represent distinct patterns of abusive and transformational leadership across leadership duos, and to investigate their differential effects on employee outcomes.

### Profiles of dual leadership

Consistent with our expectations, the analysis revealed distinct profiles of dual leadership. First, a consistently *positive profile* (53.27% of the sample) was found, which was characterized by low abusive and high transformational leadership from both leaders. Second, we found a *negative profile* (6.92%), which showed reverse patterns, reflecting a consistent but adverse leadership constellation. Third, we found two inconsistent profiles. The *inconsistent profile 1* (4.39%), was marked by high abusive supervision and moderate to low transformational leadership from the upper-level leader, while the lower-level leader exhibited low abusive and high transformational leadership. In turn, the *inconsistent profile 2* (5.68% of the sample) displayed the reverse pattern, with low abusive supervision and moderate to high transformational leadership from the upper-level leader, but high abusive supervision and low transformational leadership from the lower-level leader. Additionally, we found a fifth, *moderate positive profile* (29.74% of the sample), where both leaders engaged in moderate to low abusive supervision and moderate transformational leadership.

The first two profiles (i.e., *positive* and *negative*) as well as the *moderate positive profile* reflect consistency across leadership levels regarding both abusive and transformational behaviors. These findings are in line with previous research indicating that dual leadership is often correlated (Bormann and Diebig, 2021; Mawritz et al., 2012; Mayer et al., 2009; Rice et al., 2021). Consistent leadership patterns signal clear and coherent behavioral expectations to employees, which, in case of positive alignment, can either foster a positive work environment or, in case of negative alignment, intensify negative experiences. More importantly, the identification of two inconsistent profiles (i.e., *inconsistent profiles 1 and 2*) underlines that discrepancies or even contradictory behaviors between dual leaders are a relevant phenomenon. Specifically, leadership duos where an upper-level leader demonstrates abusive behaviors, while the lower-level leader acts transformational, and vice versa exist. This finding aligns with the existing literature, which shows that dual leaders do not always act in harmony and may diverge in their leadership approaches (Sahlmueller et al., 2022; Vidyarthi et al., 2014). These leadership inconsistencies are crucial as they highlight that employees may face conflicting leadership signals, which shape their strain and commitment. These findings replicate and extend prior research on leadership inconsistency by demonstrating that such inconsistencies are not only present within individual leaders in different situations or over time (Breevaart et al., 2014; de Cremer, 2003; Klebe et al., 2022), but also manifest between leaders across hierarchical levels.

### Associations with employee strain and commitment

The analysis revealed several important insights into how different constellations of dual leadership affect employee strain and organizational commitment. First, as expected, employees within the *positive profile* reported lower strain and higher commitment levels compared to those in the *negative profile*. This finding replicates existing evidence on the detrimental effects of negative leadership and the beneficial effects of positive leadership (e.g., Hong et al., 2024; Tautz et al., 2024), and underscores the importance of consistent positive leadership in leadership duos.

Second, employees in the *positive profile* also experienced lower strain and higher commitment than those in the *inconsistent profiles*. This indicates that the presence of only one positive leader in a dual leadership setting is insufficient to achieve the same positive outcomes as consistently positive leadership duos. It appears that the negative influence of one abusive leader undermines or neutralizes the beneficial effects of another positive leader, regardless of whether the upper- or the lower-level leader behaves positively. Alignment in positive leadership seems to be essential to fully support employee commitment and lower strain in a dual leadership setting.

Third, and most importantly, employees in the *inconsistent profiles* reported similar strain levels as those in the *negative profile*. This is in line with previous research on inconsistencies within leaders (Klebe et al., 2022; Schilling et al., 2023), and suggests that inconsistent leadership duos impact employees equally to consistently negative duos. These results indicate that discrepancies between dual leaders create uncertainty and confusion for employees (Schilling et al., 2023), undermining the positive influence of one positive leader and deteriorating the leadership climate. A similar pattern emerged for employee commitment when comparing the *inconsistent profile 2* (i.e., positive upper-level leader, negative lower-level leader) with the *negative profile*. In this case, commitment levels were equally low, suggesting that a negative lower-level leader can overshadow the effects of a positive upper-level leader. However, a notable exception was observed for *inconsistent profile 1* (i.e., negative upper-level leader, positive lower-level leader). Commitment levels were higher compared to the *negative profile*. As lower-level leaders are more likely to influence employees’ day-to-day experiences and motivation by managing immediate tasks and interpersonal relations, their behavior may be of particular importance for employee commitment.

### Interactions within dual leadership

Moreover, we expected an interaction between abusive supervision of upper- and lower-level leaders. As expected, results indicated a synergistic effect: when both leaders exhibit low levels of abusive behavior, employee strain is notably lower and commitment higher. However, these positive effects are neutralized when one of the leaders, in this case the upper-level leader, displays high abusive supervision. This pattern suggests a compounding mechanism for abusive leadership behaviors. When both leaders display non-abusive behaviors, a consistent positive leadership climate exists which accounts for better employee commitment and less strain. However, when only one out of two leaders displays abusive behavior, the positive leadership climate is undermined, leading to higher strain and lower commitment. This finding aligns with prior research indicating that consistent leadership signals across hierarchical levels are critical for employee well-being (Klebe et al., 2022; Ogunfowora, 2013; Schilling et al., 2023). Inconsistent leadership behaviors, particularly abusive leadership, may in turn irritate employees’ expectations and sense of fairness (Ogunfowora, 2013; Schilling et al., 2023), thereby intensifying strain and undermining affective commitment.

Moreover, we assumed that also the positive effect of transformational behavior of one leader would be undermined by another abusive leader. Contrary to our expectations, the interactions were non-significant. Destructive leadership behaviors are typically more salient and emotionally charged than positive behaviors, particularly when enacted by individuals in positions of higher authority (e.g., Tepper, 2000). In line with previous research (Klebe et al., 2022; Matta et al., 2017; Schilling et al., 2023), abusive leadership may therefore exert a dominant influence that overshadows the potential buffering effects of transformational leadership. From a psychological perspective, employees may assign greater weight to threatening or hostile cues than to supportive ones, consistent with negativity bias and the heightened impact of adverse social interactions. Drawing on paradox theory (Miron-Spektor et al., 2018; Smith and Lewis, 2011), such inconsistent constellations can also be understood as the simultaneous presence of contradictory, yet legitimate leadership signals. Paradox theory suggests that individuals may respond to these tensions either defensively or through integrative sensemaking. This perspective may help explain why inconsistent profiles were not necessarily more harmful than coherent negative profiles. Thus, the non-significant interaction may reflect not only the asymmetric strength of negative versus positive leadership signals but also the complex ways in which employees navigate paradoxical leadership dynamics. In such constellations, transformational behavior may not function as a compensatory resource but instead coexist with an overall deteriorated leadership climate shaped by abusive conduct. Rather than creating a protective interaction, contradictory signals from two leaders may generate ambiguity and cognitive dissonance, preventing employees from fully benefiting from transformational behaviors. Thus, the non-significant interaction may reflect the asymmetric strength of negative versus positive leadership signals rather than the absence of meaningful dynamics between leadership styles.

### Theoretical implications

The current findings highlight the critical role of dual leadership by revealing the risks posed by inconsistencies in dual leadership constellations for employee strain and commitment. Results indicate that discrepancies in dual leadership can have detrimental effects, emphasizing the dominance of abusive leadership in shaping employee experiences (Schilling et al., 2023). This underscores the importance of fostering consistency in positive leadership within dual leadership structures to support employee strain and lower commitment.

This study expands existing research on dual leadership (e.g., Litano and Morganson, 2020; Sahlmueller et al., 2022; Vidyarthi et al., 2014) by exploring the interplay of different leadership styles and their varying degrees. Moreover, it contributes to the literature on leadership inconsistencies by demonstrating that the negative effects of inconsistent leadership, previously observed within individual leaders across situations or over time (e.g., Klebe et al., 2022; Schilling et al., 2023), also manifest in dual leadership settings. Consistent with prior research (Klebe et al., 2022), the findings suggest that only a consistently positive leadership approach (i.e., high transformational and low abusive behavior) fosters employee well-being, while both inconsistent and consistently negative leadership produce similarly harmful effects. To better understand how inconsistencies in dual leadership impact employees, future research should explore underlying mechanisms such as employee contact frequency, attributions, or expectations (Klug et al., 2019).

### Practical implications

Regarding practice, the findings suggest that leadership duos should be aware of their collective influence on employee well-being. To promote consistent positive leadership in dual leadership settings, occupational health promotion should specifically target leadership duos, addressing both upper- and lower-level leaders simultaneously. Supporting leaders in acknowledging their shared responsibility for employee strain and organizational commitment is essential. For consistently negative constellations (i.e., high abusive and low transformational leadership at both levels), organizations may need to prioritize corrective interventions such as intensive leadership development, feedback-based coaching, and clear accountability structures to reduce destructive behaviors and develop transformational behaviors, at least in terms of individualized consideration and accountability and respect as part of idealized influence. In contrast, inconsistent leadership profiles require alignment-focused interventions. Joint workshops, facilitated dialogue sessions, or structured reflection exercises could help leadership duos clarify shared values, align expectations, and coordinate behavioral standards to avoid sending contradictory signals to employees. In such cases, training should emphasize perspective-taking, conflict management (Kelloway and Barling, 2010), and the development of a coherent joint leadership approach. Even in consistently positive constellations, interventions may aim to stabilize and reinforce effective collaboration, for instance through regular strategic alignment meetings or structured communication routines. At the organizational level, work design strategies that formalize coordination between leadership levels—such as clearly defined decision rights, shared goal-setting processes, and transparent communication channels—may reduce the likelihood of conflicting demands and promote sustainable, health-supportive leadership structures.

### Strengths, limitations, and recommendations for future research

This study has some limitations that should be considered when interpreting the results. First, due to the cross-sectional design, causal relationships between dual leadership and employee outcomes cannot be inferred. It is possible that employees who experience high strain or low commitment perceive their leaders more negatively. However, reverse causation alone would not account for the emergence of distinct leadership profiles and interactions. The primary aim of this study was to identify different dual leadership patterns and to examine their associations with employee strain and commitment rather than isolating causal effects of individual variables. To investigate causal effects, future research should examine dual leadership using longitudinal or diary designs. Such approaches would not only allow for stronger causal inferences but also help to determine whether dual-leadership profiles represent stable constellations or dynamic configurations that evolve over time. Moreover, longitudinal data could clarify whether the observed effects on employee strain and commitment reflect short-term reactions to specific leadership episodes or accumulate into more enduring outcomes.

Second, it would be conceivable that the relationships between leadership are influenced by common method bias (Podsakoff et al., 2003). However, Harman’s single-factor test suggests that common method bias is unlikely to have influenced the results, as a single factor accounted for only a small proportion of variance (28.24%). Additionally, it is common practice to investigate employee perceptions when examining individual outcomes (Perko et al., 2016). Since leadership can only have an impact when it is perceived by employees (Schyns and Schilling, 2013), subjective ratings remain a valuable source of data. Nevertheless, future studies would benefit from incorporating leader perspectives and additional third variables to gain deeper insights into the reciprocal dynamics between leaders and employees that may shape different leadership profiles. For example, the contact frequency between the two leaders or between each leader and their employees could play an important role in shaping these profiles and their respective effects.

Third, regarding the sample, one might argue that the military context represents a unique and highly hierarchical work environment with limited comparability to economic organizations, potentially restricting the generalizability of findings. The Armed Forces are characterized by clearly formalized authority structures and pronounced hierarchical norms, which may intensify the salience of leadership constellations and their effects on employees. However, dual leadership structures are not exclusive to the military but are also prevalent in both large and small organizations across the private and public sectors. In particular, matrix organizations, project-based structures, and cross-functional teams frequently involve employees reporting to multiple supervisors (e.g., functional and disciplinary leaders or line managers and project managers). In such contexts, leaders may likewise differ in priorities, behavioral styles, and value orientations, creating comparable patterns of alignment or divergence. At the same time, contextual characteristics such as the degree of formal hierarchy, role clarity, and cultural norms regarding authority may influence the prevalence and strength of specific dual-leadership profiles. Thus, while the structural phenomenon of dual leadership extends beyond the military, its manifestations and consequences may vary across sectors. To strengthen the external validity and the transferability of the findings, future research should aim to replicate these results with samples from various organizational contexts beyond the military.

A final limitation concerns the exclusively quantitative design of the present study. While the identified profiles provide insight into the structure and consequences of dual-leadership constellations, they do not explain why certain profiles emerge. For instance, inconsistent leadership patterns may result from limited coordination time between leaders, unclear role boundaries, divergent training backgrounds, personality differences, heightened stress, limited resources, or insufficient leadership competencies. Structural factors such as workload, organizational pressure, or competing strategic versus operational demands may also hinder alignment between hierarchical levels. Without qualitative data, it remains unclear whether inconsistencies are intentional, situationally constrained, or rooted in stable individual differences. Future research could therefore complement profile-based approaches with interviews or qualitative case studies involving leaders and employees to better understand the underlying mechanisms that give rise to consistent or inconsistent dual-leadership constellations. Quantitative follow-up studies may provide evidence for relevant antecedences.

## Conclusion

This study is the first to examine profiles and interactions within dual leadership constellations and their impact on employee strain and commitment. By considering subpopulations with varying levels of abusive and transformational leadership, the study identifies five distinct dual leadership profiles, each representing meaningful constellations of leadership behaviors at the upper and lower levels. These profiles reveal differential associations with employee strain and commitment, highlighting the critical role of leadership consistency. Both the latent profile analysis (LPA) and interaction effects suggest that dual leadership fosters employee commitment and alleviates strain *only when both leaders consistently demonstrate positive leadership* (i.e., low abusive and high transformational leadership). Importantly, the findings indicate that one leader’s positive behavior cannot compensate for the negative behavior of another, but that potential positive effects of leadership are undermined with a second negative leader. To promote and sustain employee well-being, organizations should provide targeted training and support for leadership duos, ensuring alignment in leadership approaches to foster employee commitment and lower strain.

## Data Availability

The raw data supporting the conclusions of this article will be made available by the authors, without undue reservation.
